# Functional Recovery After Alcohol Septal Ablation for Hypertrophic Obstructive Cardiomyopathy—When Measured Success Does Not Equal Complete Recovery

**DOI:** 10.3390/diagnostics16213478

**Published:** 2026-10-24

**Authors:** Ivana Burazor, Ivan Ilić

**Affiliations:** 1 National Institute for Heart and Blood Vessels Dedinje, 11040 Belgrade, Serbia; 2 Faculty of Medicine, University of Belgrade, 11040 Belgrade, Serbia

**Keywords:** hypertrophic cardiomyopathy, septal reduction therapy, alcohol septal ablation, functional testing, recovery

## Abstract

Alcohol septal ablation (ASA) is an established septal reduction therapy for symptomatic patients with hypertrophic obstructive cardiomyopathy (HOCM). Although successful treatment is traditionally defined by a reduction in left ventricular outflow tract (LVOT) obstruction and improvement in symptoms, these parameters do not necessarily indicate complete functional recovery. Persistent exercise intolerance may occur despite an apparently successful intervention, reflecting the complex pathophysiology of HCM. This review explores the mechanisms underlying incomplete functional recovery after ASA and evaluates contemporary approaches to objectively assess treatment response. Functional capacity after ASA is determined by multiple interacting factors, including residual or recurrent LVOT obstruction, abnormal mitral and papillary muscle anatomy, persistent diastolic dysfunction, myocardial fibrosis, valvular disease, pulmonary hypertension, and coronary microvascular dysfunction. Age, deconditioning, pulmonary disease, anemia, musculoskeletal limitations, and other comorbidities may independently contribute to exercise intolerance. Conventional assessment based on New York Heart Association functional class and resting echocardiography may therefore underestimate persistent physiological impairment. Cardiopulmonary exercise testing (CPET) provides a comprehensive assessment of exercise capacity by integrating cardiovascular, pulmonary, and metabolic responses. Exercise echocardiography, cardiac magnetic resonance, pulmonary function testing, and, in selected patients, invasive exercise hemodynamics provide complementary information. Emerging strategies including multimodality imaging, pharmacological therapy, individualized rehabilitation, and wearable activity monitoring may further improve assessment and long-term recovery. Conclusions: Successful reduction in LVOT obstruction after ASA should not be considered synonymous with complete functional recovery. A multimodality, physiology-oriented approach, with CPET as a central objective tool, may better identify residual limitations and guide individualized management. Future studies should focus on physiological rather than purely anatomical endpoints and determine whether patient selection, procedural precision, and phenotype-guided rehabilitation can improve functional recovery after ASA.

## 1. Introduction

Hypertrophic cardiomyopathy is one of the most common genetic anomalies of the heart, leading to a significant number of patients experiencing symptoms and requiring specific treatment. The disease itself can have different phenotypes commencing with minimal changes in the morphology of the heart without symptoms, up to severe hypertrophy with important functional impairment of the patients that are experiencing severe symptoms despite maximal medical therapy. Usually, obstruction of the left ventricular outflow tract (LVOT) causes impairment to blood flow, which is responsible for the symptoms.

Current guidelines recommend septal reduction therapy (SRT) in symptomatic patients with a gradient in LVOT greater than 50mmHg despite optimal negative inotropic therapy [1]. SRT can be either surgical myectomy or an interventional procedure, alcohol septal ablation (ASA), and the results are comparable with certain specificities for each method, like required expertise for surgical myectomy and substantial risk of permanent pacemaker implantation for ASA [2]. Most of the studies demonstrated a sustained benefit of SRT in improving patients’ functional status with change in New York Heart Association (NYHA) class, reduction in septal thickness and LVOT gradient [3,4].

However, there are patients that do not exhibit functional improvement after SRT. It has been noted that, despite successful ASA with significant reduction in septal thickness and LVOT gradient, certain patients do not improve in terms of exercise tolerance and overall quality of life [5]. The reason for this could be sought in suboptimal intervention itself, the effects of concomitant cardiovascular comorbidities or non-cardiac conditions that can affect functional capacity of a patient. HCM itself can disturb small coronary arteries comprising microcirculation, which can produce ischemia, cause arrhythmia and have a profound impact on a patient’s survival and functional capacity [6,7].

Important consideration must also be given to the actual measurement of functional recovery. Which test represents the best reflection of the effect of septal thinning and LVOT gradient reduction that is achieved with ASA? It is evident that change in New York Heart Association (NYHA) class may not provide the most accurate assessment of functional recovery after SRT. Is it the 6 min walk test (6MWT), the cardiopulmonary exercise test (CPET) or some other modality? Should evaluation be limited to noninvasive tests since there is growing evidence that invasively measured pressures during exercise in the left atrium and pulmonary circulation give more subtle information on myocardial performance in patients that would develop heart failure that can have a profound long-term impact on their survival and quality of life [8]?

In this review, we have focused on possible causes of incomplete functional recovery of patients undergoing ASA and tried to provide explanations for such outcomes in otherwise successful interventions in terms of septal thickness and LVOT gradient. We have divided the elaboration into specific segments that shed light on the possible influential factors like intervention itself, the presence of cardiovascular conditions that coexist or are caused by hypertrophic cardiomyopathy, non-cardiac conditions affecting functional capacity of an HCM patient and disturbance of microcirculation in HCM patients. We have also focused on the role of specific tests in evaluating the functional capacity of a patient with hypertrophic cardiomyopathy and provided ideas how to overcome the limitations of the tests and provide better insight into a patient’s ability and potential to recover and improve functional capacity after successful ASA.

## 2. Literature Search and Narrative Synthesis

This narrative review integrates clinical and mechanistic evidence on incomplete functional recovery following alcohol septal ablation (ASA). A narrative approach was selected because the available studies differ substantially in population, design, assessment modality, follow-up duration and outcome definitions. This heterogeneity limits direct comparisons and a meaningful pooled estimate of functional recovery.

A targeted, non-systematic PubMed/MEDLINE literature search was undertaken and updated on 9 October 2026, without a predefined publication-year restriction. Searches combined “hypertrophic cardiomyopathy” or “obstructive hypertrophic cardiomyopathy” with “alcohol septal ablation”, “septal reduction”, “functional recovery”, “exercise capacity”, “cardiopulmonary exercise testing”, “coronary microvascular dysfunction”, “coronary flow reserve”, “cardiac magnetic resonance”, or “rehabilitation”. Reference lists of relevant publications were also considered. English-language peer-reviewed original studies, observational registries, systematic reviews and meta-analyses relevant to post-ASA outcomes or functional assessment in HCM were eligible for narrative inclusion; reports without relevant clinical, physiological or functional endpoints were not prioritized. Selection was based on relevance to the review questions rather than a formal systematic screening or risk-of-bias protocol.

Evidence directly obtained in ASA populations was prioritized when discussing procedural and postprocedural effects; studies in broader HCM populations were interpreted as indirect evidence about functional capacity, disease mechanisms and rehabilitation. Studies of immediate coronary physiological responses were distinguished from longitudinal studies of symptoms or exercise performance. Representative studies, their applicability and limitations are summarized in Supplementary Table S1; its references are listed separately in the Supplementary Material.

## 3. The Influence of Intervention

The effectiveness of ASA in achieving reduction in septal thickness and decrease in LVOT gradient can be variable. It depends on several factors, and each of them can have substantial influence on treatment success. The treatment leads to different degrees of myocardial scarring in the septal territory, reduced septal thickness and LVOT enlargement with reduced impediment to blood flow which leads to LVOT gradient reduction. The initial gradient relief at the procedure itself comes from ischemia and consecutive akinesia of the basal septum caused by coronary flow obstruction achieved by balloon inflation in target septal branch of, most often, the left anterior descending (LAD) artery. Initial LVOT gradient decrease after balloon inflation can have predictive value for the outcome of entire procedure [9].

**Figure 1:**
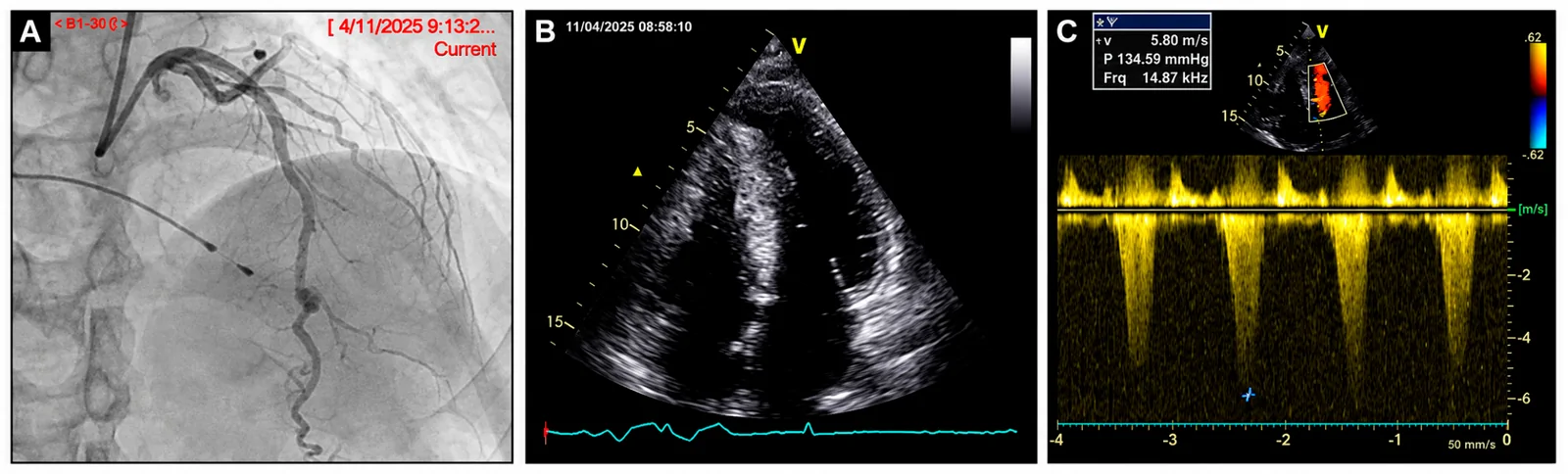
Coronary angiography of the left coronary artery demonstrating absence of septal branches suitable for alcohol septal ablation (courtesy of the Cardiology Clinic National Institute for Heart and Blood Vessels, Dedinje). (**A**) image coronary angiography of the left coronary artery PA Cranial view. (**B**) panel 2D echocardiography apical four chamber view. Depth markers indicate imaging depth in centimeters. (**C**) panel continuous wave Doppler of LVOT. Depth markers indicate imaging depth in centimeters. In the continuous-wave Doppler panel, V denotes peak flow velocity (m/s), P denotes the corresponding estimated pressure gradient (mmHg), and Freq denotes.

The anatomical distribution of coronary arteries and morphology of the left ventricle is the starting point of successful ASA. The septal branches can have variable territories and correspondence with septal myocardium, while occasionally the target septal branch cannot be identified. Besides anatomical variability, inability to identify “target” septal for ASA can often be a consequence of myocardial hypertrophy compressing the septal branches and preventing an interventionalist from spotting the one that can be treated (Figure 1). Vasodilators given intracoronary before doing coronary angiography might be helpful in selecting the appropriate branch and estimating its diameter and territory. Sometimes, the basal septum that is responsible for LVOT obstruction is supplied by a different coronary artery that cannot be easily identified. The morphology of the left ventricular cavity and anatomical relations between the LVOT, mitral and aortic valve can be altered so that when echocardiography contrast is given during ASA, the location of opacification might not be at the point of contact between the anterior septal leaflet and LVOT myocardium, which is responsible for septal anterior motion (SAM) of the anterior mitral leaflet and LVOT obstruction. However, during the procedure, this must be recognized by the absence of LVOT gradient decrease after initial artery obstruction by over-the-wire balloon (OTW) and echocardiographic contrast application. On the other hand, since the three-dimensional structure of the left ventricle is evaluated using two-dimensional echocardiography, the exact geographical location of the LVOT gradient might be missed during the procedure. This can be overcome by using 3D echocardiography to better evaluate LVOT geometry and interaction with mitral valve, as well as to precisely identify the target septal territory for alcohol application [10,11]. La Canna and associates used 3D echocardiography to classify patients into groups suitable for ASA and ones that should undergo surgical myectomy based on 3D echo contrast opacification of the septum based on echo contrast distribution in the septum [11]. Although feasible in the catheterization laboratory, 3D echocardiography requires an experienced operator, and the evidence for its use is still lacking in comparison with 2D echocardiography regarding procedural outcomes and complication when ASA is done. It would be very important to define its use for ASA and to create measurable parameters that can be used in procedural planning and execution.

Furthermore, the procedural planning can be improved by adding information about three-dimensional morphology of the left ventricle. Modern CT coronary angiography combined with 3D left ventricular modeling can provide a structure that can be analyzed and could provide quality information for the operator regarding the shape of the left ventricle, the relationship between the LVOT and anterior mitral leaflet, together with detailed distribution of potential target coronary arteries for ASA.

Due to this variability, there are still patients that do not exhibit significant gradient reduction or symptom improvement after ASA. In a large European registry, there are still around 10% of patients that did not experience the improvement in dyspnea and NYHA class. Also, the gradient reduction of more than 50mmHg was not achieved in around 10% of patients and around 5% did not experience any change in LVOT gradient [12]. These patients might be the ones that have anatomical variability in left ventricular and coronary artery morphology that made them not suitable candidates for ASA.

Another important issue is the indication for ASA compared to myectomy. The thickness of the septum above 25–30 mm in patients treated with ASA is associated with less procedural success. These patients often undergo repeated procedures, and the LVOT gradient tends to recur after initial treatment. The reason for this might be incomplete procedure in terms of LVOT gradient reduction and decrease in septal thickness [13]. These patients might be treated better with surgery, especially if additional disease of the mitral or aortic valve exists [14].

Although both ASA and surgical myectomy effectively relieve LVOT obstruction, direct comparative evidence regarding objective functional recovery after these procedures remains limited, and treatment selection should primarily reflect individual anatomical and clinical characteristics. Firoozi et al. compared 44 patients undergoing surgical myectomy or ASA and reported a significantly greater improvement in peak oxygen uptake (peak VO_2_) following myectomy (16.4 to 23.1 mL/kg/min) compared with ASA (16.2 to 19.3 mL/kg/min; *p* < 0.05 for between-group comparison), despite comparable reductions in LVOT gradient and improvements in NYHA functional class [15]. In contrast, Nagueh et al., in a comparative study of 82 patients, demonstrated significant improvements in peak VO_2_ following both surgical myectomy (18.9 to 22.2 mL/kg/min) and ASA (20.8 to 26.2 mL/kg/min), without a significant difference in functional improvement between the two procedures at one-year follow-up [16].

These findings suggest that both septal reduction techniques provide meaningful improvements in objectively measured exercise capacity, although the magnitude of improvement may vary between studies. Importantly, incomplete functional recovery may occur following surgical myectomy as well as ASA, indicating that successful relief of LVOT obstruction does not necessarily translate into normalization of exercise capacity. Therefore, both approaches appear broadly comparable in their potential to improve functional capacity, while the available evidence remains insufficient to establish consistent superiority of either technique in terms of peak VO_2_ improvement.

## 4. Influence of Cardiovascular Comorbidities

Myocardial hypertrophy is a condition that affects multiple levels of myocardial performance. Besides causing the LVOT obstruction that can produce symptoms similar to aortic stenosis like angina, dyspnea and syncope, there are more subtle changes in myocardial performance related to impaired diastolic function that can cause significant symptoms without existing LVOT obstruction.

The changes in myocardial building proteins leading to hypertrophy and subsequent fibrosis can alter diastolic properties of the left ventricle. Accompanied by changes in calcium uptake and energy metabolism due to hypertrophy, increased myocardial stiffness and disturbed relaxation are the initial signs of altered diastolic properties that can be present before LVOT obstruction develops. Depending on the genotype and phenotype of the HCM patient, diastolic dysfunction can further develop towards a restrictive filling pattern of left ventricular inflow. Diastolic dysfunction can lead to enlargement of the left atrium and increased incidence of atrial fibrillation that patients with HCM tolerate poorly [14,17]. The changes in diastolic function can have a detrimental effect on exercise capacity in patients with HCM. Several specific parameters, besides “traditional” E/e’ average, left atrial dimensions and volume, and tricuspid regurgitation velocity, like global left ventricular strain and left and right atrial strain, were found to be associated with reduced exercise capacity in HCM patients [17,18]. Su and coauthors have shown that left atrial (LA) reservoir strain correlates better than other diastolic parameters with the decrease in functional capacity of patients with HCM measured with achieved metabolic equivalents of task (METS) during treadmill exercise test [19].

Diastolic dysfunction after ASA usually improves, as has been demonstrated in several studies using follow-up echocardiography and cardiac magnetic resonance (CMR) imaging. These studies showed improved diastolic indices together with a reduction in left ventricular ejection fraction (LVEF), myocardial mass and refined indices of myocardial work, which points towards improved hemodynamics of the left ventricle after ASA [20,21]. The effects of ASA on diastolic function were comparable to the effects of the cardiac myosin inhibitor mavacamten in patients that responded to treatment with this novel medication [22]. However, not every patient responds favorably to ASA, and diastolic function does not restore to normal after intervention. This must be accounted for when evaluating functional capacity of HCM patients after successful ASA.

**Figure 2:**
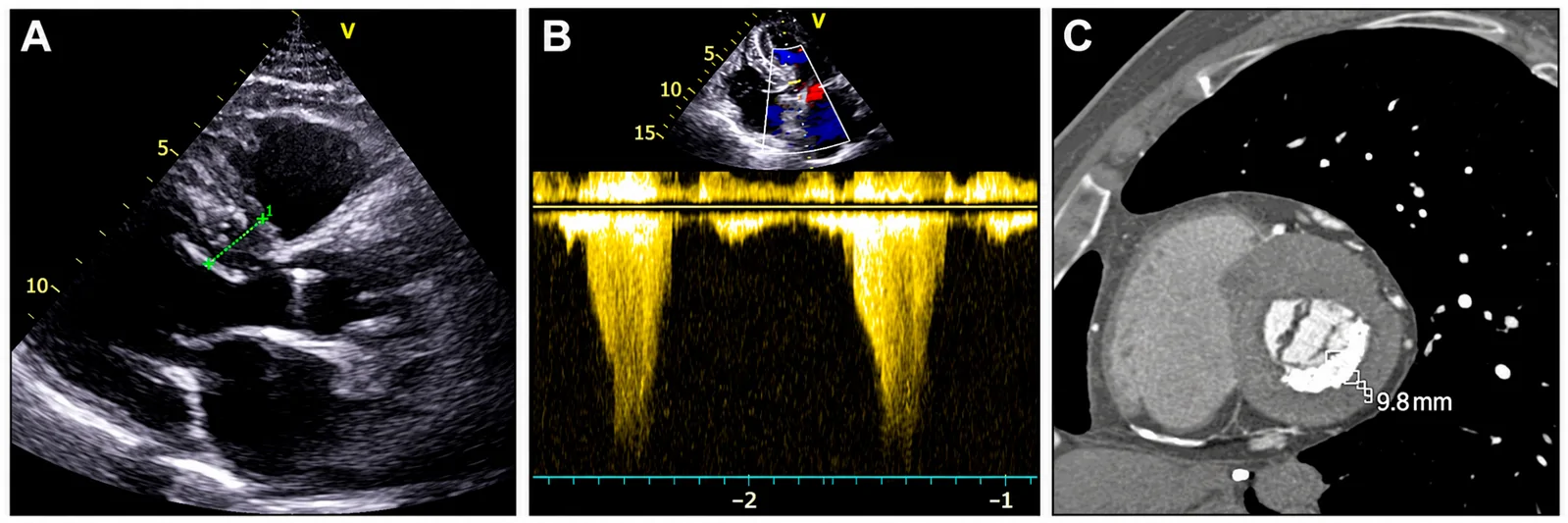
CT and echocardiography images of a patient with hypertrophic obstructive cardiomyopathy and severe calcifications of mitral annulus deemed unsuitable for valve surgery (courtesy of the Cardiology Clinic National Institute for Heart and Blood Vessels, Dedinje). (**A**): Echocardiographic image showing the left ventricular outflow tract and septal measurement. (**B**): Color Doppler and spectral Doppler images demonstrating flow acceleration and obstruction in LVOT. (**C**): Cardiac CT demonstrating extensive mitral annular calcification, with a displayed measurement of 9.8 mm.

Quite often there are coexisting valve abnormalities that accompany myocardial hypertrophy. Sometimes it is difficult to discern true obstructive HCM with significant resting gradient in LVOT and degenerative aortic valve disease. In older patients, degenerative valve disease coexists with HCM, and treatment has to be tailored according to the degree of valve degeneration [13,14]. The diagnostic pathway should include a CT scan of the aortic valve and measurement of calcium score, which can help in elucidating degenerative valve disease, and in such patients, surgery that includes septal myectomy and valve reconstruction might be a better option. Sometimes, the patient’s frailty will play a role regarding treatment despite advanced valve disease. Coexisting obstructive HCM and significant valve disease poses a prohibitive risk in frail patients with multiple comorbid conditions. This is very often the case in degenerative mitral valve disease producing severe mitral insufficiency that cannot be treated surgically due to extensive calcification of the valve annulus (Figure 2) [13]. The patient described in Figure 2 underwent ASA due to prohibitive risk of surgery with the acceptable interventional result. However, a patient like this cannot be expected to achieve functional recovery like patients that do not have valve disease.

If we move away from these extremes, there are still patients that have mitral leaflet abnormalities that can affect the outcome of ASA. Anterior mitral valve leaflet length, and its relationship with the septum or left ventricular cavity, can be predictive of the degree of LVOT obstruction. Also, papillary muscle insertion and chordal abnormalities can profoundly affect the development of septal hypertrophy, thus adding complexity to percutaneous treatment of LVOT obstruction [23,24]. These parameters can significantly impact the success of ASA, as was found in a pilot study by Coifan and associates. This study included little more than 30 patients and found that mitral valve morphological features like redundant anterior mitral leaflet, defined as excessive leaflet tissue protruding towards the LVOT, greater posterior mitral leaflet projection (length from the base of the leaflet to the coaptation point) and anterior displacement of the coaptation point between the leaflets were associated with suboptimal response to ASA [25]. Incomplete reduction in LVOT gradient can still cause symptoms, especially during exercise or provocation, which can be documented at follow-up echocardiography examination after ASA.

Atrial fibrillation represents an important contributor to impaired functional capacity in patients with HCM, particularly in the presence of diastolic dysfunction and left atrial remodeling. Azarbal et al. demonstrated significantly reduced exercise capacity in HCM patients with paroxysmal AF, even when sinus rhythm was present during CPET assessment. Therefore, AF should be considered as a potential determinant of persistent functional limitation following otherwise successful ASA, emphasizing the importance of rhythm assessment during follow-up [14,26].

Hypertrophic cardiomyopathy has been associated with disturbance of coronary microcirculation [27]. It has been shown that even genotype-positive and phenotype-negative patients who are carriers of genes associated with HCM have disturbed myocardial metabolism in terms of depleted adenosine triphosphate (ATP) reserve in mitochondria, which can affect the function of sarcomeres carrying the mutation [28]. Abnormal myocardial hypertrophy is associated with capillary rarefaction and adverse remodeling of intramural coronary arterioles—specifically, medial hypertrophy and increased wall-to-lumen ratio—resulting in impaired coronary flow and microvascular dysfunction throughout the left ventricle. Coronary microvascular dysfunction (CMD) has been associated with ischemia that can further promote myocardial fibrosis, which can have multiple consequences. Ischemia and fibrosis have profound prognostic impact in HCM and can lead to development of left ventricular remodeling and heart failure, malignant arrhythmias and sudden cardiac death [6,7]. The degree of CMD can be reliably evaluated noninvasively using positron emission tomography (PET), which can detect CMD in HCM [6]. However, PET is not readily available for most HCM patients. Unfortunately, exercise stress test has limited sensitivity to detect myocardial ischemia in these patients [29].

Currently, invasive coronary physiology, using the thermodilution technique and coronary guidewire to measure pressure and temperature, can offer insight into the status of microcirculation and assign patients with angina and exercise intolerance into several endotypes based on structural or functional disturbance of small coronary vessels [30]. Although this examination became standard for patients with ischemia with no obstructive coronary arteries (INOCA), HCM patients have not been studied extensively using this method. However, there are studies that show that disturbance in microcirculation is expressed as a decrease in coronary flow reserve (CFR), mostly due to increased resting coronary blood flow, although in patients with long-standing disease it can be expected that changes occurred at structural levels of microcirculation, causing increased microvascular resistance [7,31]. On the other hand, it would be expected that reversal of obstruction by ASA would cause an immediate improvement in microcirculation in HCM patients. This would be especially true if the disturbance is mainly caused by increased resting flow. This was proven by studies from Jabber using Doppler coronary wire and by Terauchi using the thermodilution method and coronary guidewire capable of measuring pressure and temperature. Both studies showed improvement in CFR and conflicting results regarding microvascular resistance, where there was an increase in coronary resistance in the Doppler wire study, while there was a decrease in coronary resistance expressed as index of microcirculatory resistance (IMR) in the thermodilution study [32,33]. It would be expected that due to resolution of LVOT obstruction, an immediate rise in CFR should be accompanied by a decrease in resistance in small vessels. However, ASA induces localized septal myocardial infarction, which may transiently affect coronary microvascular function and potentially attenuate the beneficial effects of LVOT obstruction relief [34]. The immediate microvascular response may therefore reflect competing mechanisms, including improved coronary hemodynamics following obstruction relief, and potential adverse effects of procedure-related myocardial injury cause myocardial infarction, which, by itself, can provoke CMD and blunt the response to successful relief of LVOT gradient [34].

In our recently published invasive physiology study in HCM patients undergoing ASA without previous coronary artery disease (CAD), we performed absolute flow (Q) thermodilution measurements before and after septal ablation. This allows measurement of Q, CFR and microvascular resistance (R) using a method that is less operator-dependent than bolus thermodilution. We found that CFR improved in patients that already had decreased CFR below 2.0 prior to the procedure, while those that had normal CFR before experienced a decrease in its value after successful ASA. These divergent responses may reflect the balance between improved coronary hemodynamics following LVOT obstruction relief and potential adverse effects of procedure-related myocardial injury. However, the contribution of these mechanisms remains hypothetical and requires further investigation. Importantly, the longer-term evolution of microvascular function over the subsequent 3–6 months after ASA remains insufficiently characterized [35]. Based on this data, the degree of disturbance of microcirculation is difficult to assess in patients with HCM; it is possible it depends on the duration of the disease itself, concomitant atherosclerosis and degree of myocardial fibrosis. CMR may provide complementary information on coronary microvascular dysfunction and myocardial remodeling in HCM. Stress perfusion imaging, including quantitative perfusion mapping, can identify myocardial perfusion abnormalities, whereas late gadolinium enhancement (LGE) and extracellular volume (ECV) assessment characterize focal and diffuse myocardial fibrosis, respectively. Although these parameters provide diagnostic and prognostic information in HCM, their ability to predict microvascular and functional recovery specifically after ASA remains insufficiently established. The effect of intervention also can be quite unpredictable, and residual CMD can profoundly affect the functional capacity of the patient undergoing percutaneous SRT. Combined studies using CMR and invasive assessment of microcirculation could be able to predict the outcome of ASA regarding microcirculation.

**Figure 3:**
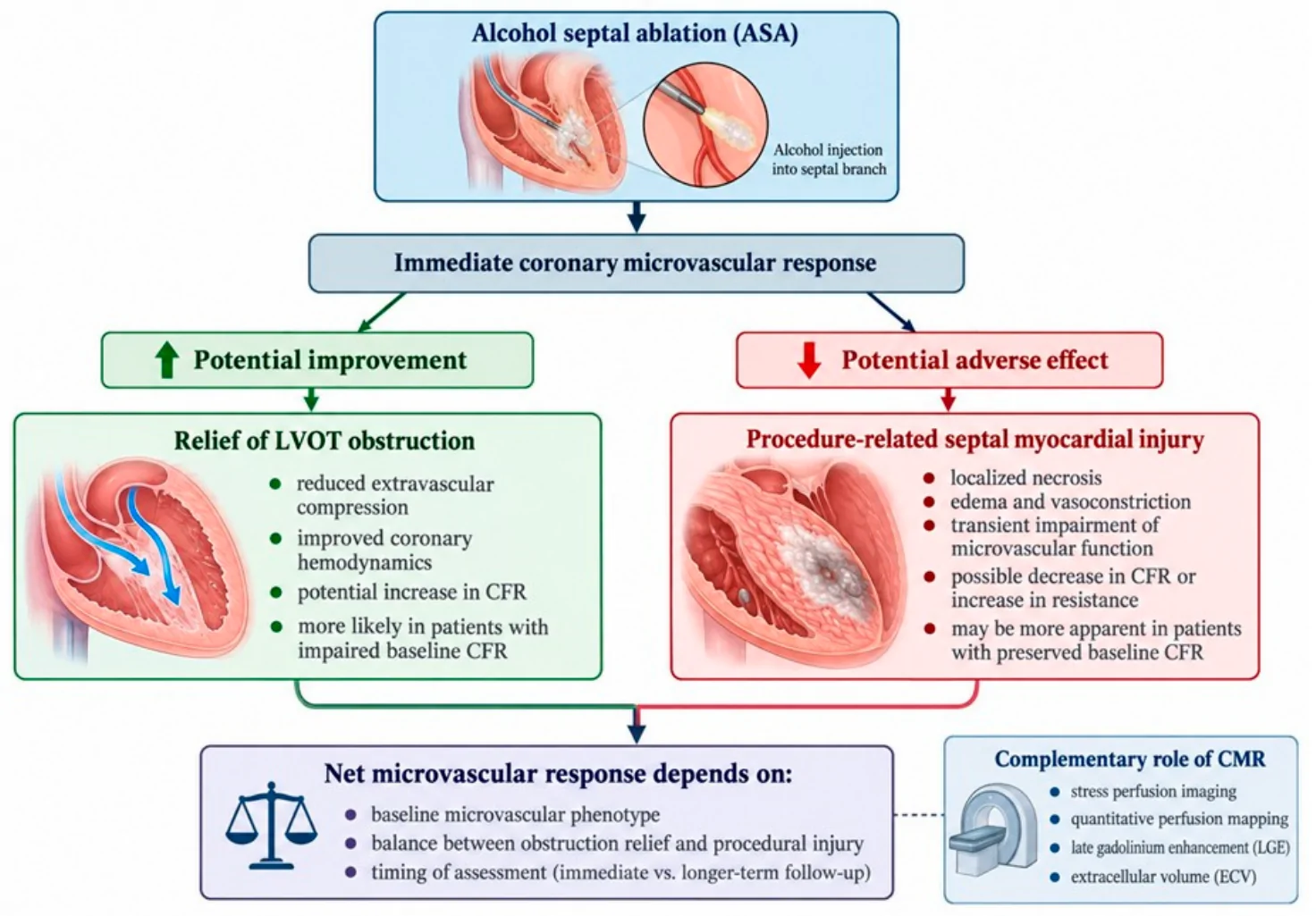
Proposed mechanisms of heterogeneous coronary microvascular responses after alcohol septal ablation.

From a clinical perspective, coronary microvascular dysfunction should be considered in HCM patients with persistent angina or exercise intolerance, particularly when symptoms remain unexplained by the degree of LVOT obstruction or coexist despite successful septal reduction. Following the exclusion of relevant epicardial coronary artery disease and assessment of other potential causes of symptoms, quantitative myocardial perfusion imaging using PET or stress CMR may provide valuable information on microvascular function. In selected patients with persistent symptoms or inconclusive noninvasive findings, invasive coronary physiological assessment using CFR and microvascular resistance measurements may offer additional insights into the underlying mechanisms of CMD. However, the interpretation of these measurements in HCM requires caution, as reduced CFR may reflect increased resting coronary blood flow, impaired hyperemic vasodilation, or a combination of both. Importantly, the clinical utility of routine invasive CMD assessment after ASA and its implications for treatment selection and functional recovery remain to be established (Figure 3).

## 5. Functional Testing to Evaluate Recovery After ASA

Assessment of functional recovery after ASA is challenging because exercise capacity in HCM is determined by several interacting mechanisms. A meaningful assessment should therefore capture not only symptom perception but also the patient’s physiological response to exercise.

NYHA functional class remains clinically useful but is inherently subjective. It is influenced by patient perception, activity level, motivation, and adaptation to chronic symptoms. The 6MWT provides a simple measure of submaximal exercise performance and may be useful when CPET is unavailable, but it is also affected by age, body habitus, musculoskeletal limitations, deconditioning, and motivation.

CPET provides a more comprehensive assessment because it simultaneously evaluates oxygen uptake, ventilation, heart rate, blood pressure, and exercise workload. Relevant parameters include peak oxygen uptake (peak VO_2_), percentage of predicted peak VO_2_, oxygen uptake at the anaerobic threshold, oxygen pulse, ventilatory efficiency (VE/VCO_2_ slope), chronotropic response, blood pressure response, and heart-rate recovery.

The value of CPET in HCM is supported by several lines of evidence. In a cohort of 156 HCM patients, Finocchiaro et al. demonstrated that peak cardiac index was the principal determinant of peak VO_2_, while age, sex, right ventricular parameters, and diastolic variables also contributed to exercise capacity. Reduced peak VO_2_, impaired ventilatory efficiency, and increased left atrial volume were associated with adverse clinical outcomes [36]. These findings emphasize that exercise intolerance in HCM is multifactorial rather than simply a consequence of LVOT obstruction.

Several systematic reviews and meta-analyses involving large HCM populations have consistently demonstrated the prognostic relevance of CPET-derived parameters, particularly reduced peak VO_2_, impaired ventilatory efficiency, abnormal blood pressure response, and heart-rate recovery [37,38,39]. These findings support the value of CPET in evaluating functional impairment in HCM, although their direct applicability to functional recovery following ASA remains to be established.

Most importantly for the present discussion, ASA-specific evidence supports serial CPET assessment. Rigopoulos et al. evaluated 21 patients before and three months after ASA. Patients who subsequently demonstrated clinical improvement had significant improvements in peak VO_2_, percentage predicted VO_2_, workload, ventilatory parameters, heart-rate response, and systolic blood pressure, whereas non-responders did not demonstrate comparable changes. The authors proposed CPET as an early marker of clinical success after ASA [5]. Although the study was small, its design directly addresses the question of functional recovery after septal ablation.

Although CPET provides a comprehensive and objective assessment of exercise capacity, its role as the preferred modality for evaluating functional recovery after ASA has not been sufficiently validated. Current evidence is largely extrapolated from broader HCM populations, while ASA-specific studies remain limited in number and sample size. Therefore, a multimodal approach integrating CPET with exercise echocardiography, advanced cardiac imaging, and, in selected cases, invasive exercise hemodynamics may provide a more comprehensive assessment of postprocedural recovery. Future prospective studies are needed to identify and validate the most reliable functional endpoints following ASA.

Exercise echocardiography provides complementary information. Whereas CPET quantifies the physiological consequence of exercise limitation, exercise echocardiography can identify dynamic LVOT obstruction, exercise-induced mitral regurgitation, pulmonary hypertension, and changes in filling pressures. This may be particularly useful in patients whose resting echocardiogram appears satisfactory but whose symptoms occur predominantly during exercise [14].

Advanced imaging may further explain persistent limitations. CMR can characterize myocardial hypertrophy, fibrosis, ventricular volumes, deformation, and mitral or papillary muscle anatomy, while multimodality imaging can integrate these findings with coronary and LVOT anatomy [18,20].

In selected patients with unexplained severe exercise intolerance, invasive exercise hemodynamics may provide additional information. Simultaneous assessment of pulmonary artery and left-sided filling pressures can identify abnormal hemodynamic responses that are not apparent at rest. The integration of CPET with hemodynamic measurements may therefore be particularly valuable when symptoms are disproportionate to conventional echocardiographic findings [40].

Pulmonary function testing may also improve interpretation of CPET. A recent prospective study of 102 HCM patients demonstrated significant associations between forced vital capacity and FEV_1_ and between peak VO_2_ and peak ventilation, suggesting that pulmonary function testing can complement CPET when the pulmonary contribution to exercise limitation is uncertain [41].

Finally, laboratory-based exercise testing does not necessarily reflect real-world physical activity. Recent accelerometry data demonstrates that HCM patients may have lower daily step counts, less moderate-to-vigorous activity, and greater sedentary time, with physical activity independently related to quality of life [42]. Thus, future assessments of recovery should distinguish between exercise capacity measured in the laboratory and physical activity performed in everyday life.

The selection of complementary diagnostic modalities should be individualized according to the suspected mechanisms of persistent functional limitation after ASA.

## 6. Influence of Non-Cardiac Conditions

Functional recovery after ASA cannot be interpreted exclusively through the cardiovascular phenotype. Age, physical conditioning, pulmonary function, body composition, medication, anemia, renal disease, metabolic disorders, and musculoskeletal limitations may all influence measured exercise capacity.

Age is an important determinant of exercise performance even in otherwise healthy individuals and remains relevant in HCM. In studies of HCM populations, age has repeatedly been associated with lower exercise capacity [36,37,38]. Consequently, interpretation of peak VO_2_ should rely on age- and sex-adjusted predicted values rather than absolute values alone.

Medication may also modify exercise responses. Beta-blockers and other negative chronotropic or inotropic therapies may reduce peak heart rate and alter peak VO_2_ without necessarily indicating treatment failure. This is another reason why multiple CPET parameters should be interpreted together rather than relying on a single numerical threshold [36,37].

Physical inactivity and deconditioning represent important non-cardiac contributors to persistent functional limitation in patients with HCM. Symptom-related activity restriction and fear of exercise may promote skeletal muscle deconditioning, further reducing exercise tolerance despite successful relief of LVOT obstruction. These factors should be considered when evaluating incomplete functional recovery after ASA [43].

Pulmonary and systemic comorbidities may substantially limit exercise capacity independently of successful ASA. Obstructive or restrictive pulmonary disease, impaired ventilatory reserve, anemia, iron deficiency, chronic kidney disease, diabetes, and obesity can contribute to persistent functional impairment through different physiological mechanisms. Pulmonary function testing may complement CPET when respiratory limitations are suspected [41]. Musculoskeletal disorders, peripheral vascular disease, and neuromuscular conditions may additionally restrict exercise performance or cause premature termination of exercise testing. Recognition of these conditions is essential for distinguishing cardiac from non-cardiac causes of incomplete functional recovery.

Psychological and behavioral factors should also be considered. Anxiety, fear of exercise, and reduced confidence following a cardiac diagnosis may influence both laboratory exercise performance and habitual physical activity. Objective assessment of daily activity may therefore complement conventional exercise testing [42].

From a practical perspective, persistent functional limitation after technically successful ASA can be evaluated using a pragmatic framework comprising three principal categories of potential contributing factors: (1) **HCM-related mechanisms**, including residual or recurrent LVOT obstruction, persistent diastolic dysfunction, myocardial fibrosis, and coronary microvascular dysfunction; (2) **concomitant cardiovascular conditions**, such as coronary artery disease, valvular heart disease, atrial fibrillation, and pulmonary hypertension not primarily attributable to HCM; and (3) **non-cardiac conditions**, including pulmonary disease, anemia, metabolic disorders, musculoskeletal limitations, and physical deconditioning.

This classification is based on the pathophysiological mechanisms contributing to exercise intolerance rather than on a previously validated diagnostic model or prospectively derived criteria. Its purpose is to provide a simple and clinically applicable approach to identifying potential causes of incomplete functional recovery and guiding further diagnostic investigations. Importantly, these categories are not mutually exclusive, and a **mixed phenotype**, involving more than one contributing mechanism, may frequently occur in patients with HCM. This framework also emphasizes that further reduction in the LVOT gradient alone is unlikely to normalize functional capacity when other cardiac or non-cardiac mechanisms contribute substantially to exercise limitation.

A provisional stepwise approach may help identify these contributing mechanisms. Initial assessment should include clinical evaluation, resting echocardiography, and screening for relevant comorbidities. In patients with persistent exercise limitations despite satisfactory resting LVOT gradient reduction, CPET and comprehensive resting and exercise echocardiography may help identify factors contributing to impaired functional recovery. HCM-related mechanisms may be suspected in the presence of residual provocable obstruction, persistent diastolic dysfunction, or evidence of impaired cardiac functional reserve. Concomitant cardiovascular conditions, including valvular disease, coronary artery disease, or atrial fibrillation, should also be considered. Non-cardiac causes may be suspected when cardiac findings are satisfactory and exercise intolerance is primarily attributable to pulmonary disease, deconditioning, musculoskeletal limitations, or systemic comorbidities. In patients with more than one relevant contributing factor, a mixed phenotype should be considered. Advanced imaging, coronary physiological assessment, or invasive exercise hemodynamics should be reserved for selected patients with unexplained or discordant findings. This proposed classification is intended as a practical clinical framework rather than a validated diagnostic algorithm.

## 7. How to Improve Outcomes—Future Directions

Future research should focus on improving patient selection, optimizing procedural strategies, and establishing objective functional endpoints to better predict and evaluate recovery following ASA.

### Patient Selection and Timing

Appropriate patient selection remains essential for achieving meaningful functional recovery after ASA. Beyond conventional anatomical criteria, myocardial fibrosis, diastolic dysfunction, coronary microvascular abnormalities, and associated cardiovascular conditions may influence the potential for recovery [1,13,14,18,23,24,25]. Whether earlier intervention, before the development of advanced myocardial and atrial remodeling, improves long-term functional outcomes remains an important question for future prospective studies.

## 8. Contemporary Pharmacological Therapy

Cardiac myosin inhibitors, particularly mavacamten, have changed the therapeutic landscape of obstructive HCM. In patients with persistent symptoms despite first-line medical therapy, cardiac myosin inhibitors represent an additional treatment option alongside disopyramide and septal reduction therapy, with treatment selection guided by individual clinical characteristics, anatomical suitability, patient preferences, and expertise at specialized HCM centers. Their ability to reduce hypercontractility and LVOT obstruction without producing a targeted myocardial infarction distinguishes them mechanistically from ASA [22]. Although its potential role in patients with residual symptoms after ASA is clinically relevant, evidence supporting this approach remains limited. Further studies are needed to determine whether myosin inhibitors can improve functional recovery in patients with persistent symptoms following septal reduction. Comparative studies should therefore evaluate not only gradient reduction but also changes in peak VO_2_, ventilatory efficiency, myocardial energetics, diastolic reserve, CMD, fibrosis, and quality of life.

An important unanswered question is whether pharmacological reduction in obstruction before ASA could improve patient selection or alter the extent of septal ablation required. Conversely, patients with residual symptoms after ASA may represent a population in whom adjunctive pharmacological therapy could be valuable, although this strategy requires further clinical investigation.

## 9. Procedural Precision

Advanced multimodality imaging and computational approaches may improve individualized procedural planning and anatomical targeting during ASA [10,11,18,23]. Future studies should determine whether these strategies improve not only procedural success and LVOT gradient reduction but also long-term functional recovery.

### Functional Recovery as a Procedural Endpoint

Future prospective studies should incorporate objective functional endpoints alongside conventional measures of procedural success, including LVOT gradient reduction and symptomatic improvement. Serial assessment of exercise capacity, complemented by targeted physiological and imaging evaluation, may help determine whether successful septal reduction translates into sustained functional recovery. However, standardized and validated ASA-specific functional endpoints remain to be established [5,36].

Reduced peak VO_2_ (e.g., <80% of predicted), impaired ventilatory efficiency (elevated VE/VCO_2_ slope), chronotropic incompetence, and abnormal exercise-induced pulmonary pressure responses may suggest incomplete functional recovery; however, these findings are not specific to residual dysfunction after ASA, and no validated ASA-specific CPET thresholds currently exist.

Exercise echocardiography may provide complementary information regarding residual or provocable LVOT obstruction and exercise-related hemodynamic abnormalities after ASA. Long-term follow-up data supports the sustained reduction in provocable LVOT obstruction after ASA, evaluated by exercise echocardiography, with Kashtanov et al. reporting a decrease in post-exercise LVOT gradient from 113 mmHg at baseline to 38 mmHg at one year and 29 mmHg at ten years [44]. In a prospective study of 24 patients, Dybro et al. demonstrated significant reductions in LVOT gradients during and immediately after exercise, together with lower exercise-induced pulmonary artery wedge pressures approximately seven months following ASA. Patients experienced functional improvement in terms of lower NYHA class and better score on the Kansas City Cardiomyopathy Questionnaire (KCCQ). Importantly, these improvements were not accompanied by a significant increase in peak oxygen consumption, suggesting that relief of obstruction and improved exercise hemodynamics do not necessarily translate into complete restoration of functional capacity. These findings support the complementary role of exercise echocardiography and CPET in evaluating recovery after ASA [40]. Invasive exercise hemodynamic assessment may be considered in selected patients with persistent exercise intolerance after ASA when noninvasive investigations provide inconclusive or discordant findings. Assessment of exercise-induced changes in pulmonary artery wedge pressure, pulmonary pressures, and cardiac output may help identify residual hemodynamic abnormalities and guide further management.

## 10. Rehabilitation After Septal Reduction

Exercise rehabilitation represents a promising but insufficiently investigated strategy for improving functional recovery after ASA. Evidence from broader HCM populations suggests that structured exercise training may improve functional capacity in appropriately selected patients [43]. Wasserstrum et al. demonstrated an improvement in exercise capacity from 5.3 to 6.7 METs following rehabilitation, without necessarily corresponding changes in NYHA class or conventional echocardiographic parameters [43]. Recent systematic reviews and meta-analyses further support the potential benefits and safety of individualized exercise training in HCM, while emphasizing the heterogeneity and limitations of available evidence [45,46]. However, these findings cannot be directly extrapolated to patients following ASA.

Nevertheless, phenotype-guided rehabilitation after ASA remains a proposed strategy, as prospective evidence supporting its efficacy and safety in this specific population is currently limited. Patients with predominantly cardiac limitation may require a different strategy from those with deconditioning or peripheral limitations. CPET could serve not only as an outcome measure but also as a tool for prescribing and adjusting exercise intensity. Future prospective studies should investigate whether phenotype-guided rehabilitation, incorporating residual LVOT obstruction, arrhythmic risk, CPET-derived limitations, and relevant comorbidities, can improve objective exercise capacity and quality of life after septal reduction.

## 11. From Laboratory Capacity to Real-World Recovery

Although objective exercise testing provides valuable information on functional capacity, it may not fully reflect physical activity in everyday life. Wearable devices, including research-grade accelerometers (e.g., ActiGraph) and commercially available smartwatches or activity trackers (e.g., Apple Watch, Garmin, and Fitbit), may provide complementary information on daily step counts, activity intensity, sedentary time, and heart rate. Observational studies using accelerometry in HCM have demonstrated associations between daily step counts, LVOT obstruction, symptom burden, and quality of life [42,47]. However, evidence regarding the validity and reproducibility of these measurements specifically in HCM remains limited, and validated disease-specific reference values are lacking. In clinical practice, periodic monitoring of selected activity metrics, rather than continuous collection of extensive physiological data, may offer a feasible approach to assessing changes in real-world physical activity following ASA. Future longitudinal studies should determine whether improvements in exercise capacity after ASA translate into sustained increases in real-world physical activity and quality of life.

## 12. Conclusions

Alcohol septal ablation is an effective treatment for appropriately selected patients with obstructive hypertrophic cardiomyopathy. However, successful reduction in LVOT obstruction does not necessarily result in complete functional recovery. Persistent exercise limitation may reflect the interaction of residual cardiac abnormalities, coronary microvascular dysfunction, myocardial fibrosis, arrhythmias, and non-cardiac comorbidities, emphasizing the limitations of evaluating treatment success solely through resting echocardiography and NYHA functional class.

A comprehensive, phenotype-oriented approach integrating objective functional testing, multimodality imaging, and selected physiological assessment may improve the identification of mechanisms responsible for incomplete recovery. Although CPET provides valuable integrative information, further prospective studies are needed to establish validated functional endpoints after ASA and to determine whether individualized diagnostic strategies and rehabilitation can improve long-term patient outcomes.

## Data Availability

No new data were created or analyzed in this study. Data sharing is not applicable to this article.

## Supplementary Materials

The following supporting information can be downloaded at: https://www.mdpi.com/article/10.3390/diagnostics16213478/s1. Table S1: Selected evidence relevant to functional recovery after alcohol septal ablation. Representative publications identified through targeted PubMed/MEDLINE searching and reference screening. Studies are grouped by direct relevance to the ASA population versus indirect evidence from broader HCM cohorts. The table is selective and does not represent an exhaustive systematic evidence inventory.

## Abbreviations

The following abbreviations are used in this manuscript:

ASA	Alcohol septal ablation
CFR	Coronary flow reserve
CMD	Coronary microvascular dysfunction
CMR	Cardiac magnetic resonance
CPET	Cardiopulmonary exercise testing
HCM	Hypertrophic cardiomyopathy
IMR	Index of microcirculatory resistance
INOCA	Ischemia with no obstructive coronary arteries
LA	Left atrium
LAD	Left anterior descending
LVOT	Left ventricular outflow tract
METS	Metabolic equivalents of task
NYHA	New York Hear Association
PET	Positron emission tomography
SAM	Septal anterior motion
SRT	Septal reduction therapy
6MWT	Six-minute walk test
