# Supplementary material for: Functional Recovery After Alcohol Septal Ablation for Hypertrophic Obstructive Cardiomyopathy—When Measured Success Does Not Equal Complete Recovery
Source: Diagnostics (Basel). 2026 Oct 24;16(21):3478. doi: 10.3390/diagnostics16213478 (PMC13761871; doi:10.3390/diagnostics16213478)
Supplement: Supplementary file 1 [file diagnostics-16-03478-s001.zip › diagnostics-4581783-supplementary.pdf]

Supplementary Material

Table S1. Selected evidence relevant to functional recovery after alcohol septal ablation. Representative publications identified through targeted PubMed/MEDLINE searching and reference screening. Studies are grouped by direct relevance to the ASA population versus indirect evidence from broader HCM cohorts. The table is selective and does not represent an exhaustive systematic evidence inventory.

Study	Population / follow-up	Method / endpoint	Interpretation / limitations
**A. Direct ASA population evidence**			
Rigopoulos et al. [S1]	21; pre-ASA and 3 months	Serial CPET; later clinical outcome	CPET changes associated with clinical response; small ASA-specific sample
Jaber et al. [S2]	7 underwent ASA; immediate	Doppler CFR and coronary resistance	Immediate physiological changes, not longitudinal functional recovery
Terauchi et al. [S3]	Obstructive HCM undergoing ASA; peri-procedural	Invasive microvascular indices	Microvascular response, not a validated post-ASA exercise endpoint
Misevic et al. [S4]	23; pre- and immediately post-ASA	Continuous thermodilution: Q, CFR, R	Variable individual microvascular responses; no serial functional testing
Veselka et al. [S5]	1,275 ASA patients; median 5.7 years	NYHA class, LVOT gradient, survival	Clinical long-term outcomes; not serial CPET
**B. Indirect evidence from broader HCM populations**			
Finocchiaro et al. [S6]	156 HCM patients	CPET and comprehensive exercise physiology	Functional determinants outside post-ASA setting
Bayonas-Ruiz et al. [S7]	Systematic review/meta-analysis in HCM	CPET outcomes	Indirect exercise-function evidence, heterogeneous cohorts
Rodrigues et al. [S8]	Systematic review in HCM	Exercise testing and prognosis	Indirect prognostic evidence, not intervention response
Wasserstrum et al. [S9]	HCM rehabilitation cohort	Exercise rehabilitation, METs, NYHA	Functional capacity can improve; not post-ASA specific
Cecchi et al. [S10]	HCM prognostic cohort	PET coronary microvascular function	Mechanistic/prognostic CMD evidence; not post-ASA

Abbreviations: ASA, alcohol septal ablation; CFR, coronary flow reserve; CMD, coronary microvascular dysfunction; CPET, cardiopulmonary exercise testing; HCM, hypertrophic cardiomyopathy; LVOT, left ventricular outflow tract; METs, metabolic equivalents; NYHA, New York Heart Association; PET, positron emission tomography; Q, absolute coronary blood flow; R, microvascular resistance.

References for Table S1

S1. Rigopoulos AG, Panou F, Sakadakis E, et al. Cardiopulmonary exercise test parameters at three months after alcohol septal ablation in hypertrophic obstructive cardiomyopathy are associated with late clinical outcome. Heart Lung Circ. 2020;29(2):202-210. doi:10.1016/j.hlc.2018.12.007.

S2. Jaber WA, Yang EH, Nishimura RA, et al. Immediate improvement in coronary flow reserve after alcohol septal ablation in patients with hypertrophic obstructive cardiomyopathy. Heart. 2009;95(7):564-569. doi:10.1136/hrt.2008.148239.

S3. Terauchi T, Hiraya D, Usami K, et al. A pilot study on coronary microvascular dysfunction in obstructive hypertrophic cardiomyopathy: impact of percutaneous transluminal septal myocardial ablation. Cardiovasc Interv Ther. 2025;40(4):973-981. doi:10.1007/s12928-025-01154-1.

S4. Misevic VV, Golocevac AN, Krupnikovic KS, et al. Coronary flow in hypertrophic obstructive cardiomyopathy-immediate effects of alcohol septal ablation. Catheter Cardiovasc Interv. Published online July 30, 2026. doi:10.1002/ccd.70791.

S5. Veselka J, Jensen MK, Liebregts M, et al. Long-term clinical outcome after alcohol septal ablation for obstructive hypertrophic cardiomyopathy: results from the Euro-ASA registry. Eur Heart J. 2016;37(19):1517-1523. doi:10.1093/eurheartj/ehv693.

S6. Finocchiaro G, Haddad F, Knowles JW, et al. Cardiopulmonary responses and prognosis in hypertrophic cardiomyopathy: a potential role for comprehensive noninvasive hemodynamic assessment. JACC Heart Fail. 2015;3(5):408-418. doi:10.1016/j.jchf.2014.11.011.

S7. Bayonas-Ruiz A, Muñoz-Franco FM, Ferrer V, et al. Cardiopulmonary exercise test in patients with hypertrophic cardiomyopathy: a systematic review and meta-analysis. J Clin Med. 2021;10(11):2312. doi:10.3390/jcm10112312.

S8. Rodrigues T, Cavaco Raposo S, Brito D, Lopes LR. Prognostic relevance of exercise testing in hypertrophic cardiomyopathy: a systematic review. Int J Cardiol. 2021;339:83-92. doi:10.1016/j.ijcard.2021.06.051.

S9. Wasserstrum Y, Barbarova I, Lotan D, et al. Efficacy and safety of exercise rehabilitation in patients with hypertrophic cardiomyopathy. J Cardiol. 2019;74(5):466-472. doi:10.1016/j.jjcc.2019.04.013.

S10. Cecchi F, Olivotto I, Gistri R, Lorenzoni R, Chiriatti G, Camici PG. Coronary microvascular dysfunction and prognosis in hypertrophic cardiomyopathy. N Engl J Med. 2003;349(11):1027-1035. doi:10.1056/NEJMoa025050.
